# Lower *ADD1* Gene Promoter DNA Methylation Increases the Risk of Essential Hypertension

**DOI:** 10.1371/journal.pone.0063455

**Published:** 2013-05-15

**Authors:** Li-Na Zhang, Pan-Pan Liu, Lingyan Wang, Fang Yuan, Leiting Xu, Yanfei Xin, Li-Juan Fei, Qi-Long Zhong, Yi Huang, Limin Xu, Ling-Mei Hao, Xu-Jun Qiu, Yanping Le, Meng Ye, Shiwei Duan

**Affiliations:** 1 Zhejiang Provincial Key Laboratory of Pathophysiology, School of Medicine, Ningbo University, Ningbo, Zhejiang, China; 2 Bank of Blood Products, Ningbo No. 2 Hospital, Ningbo, Zhejiang, China; 3 State Key Laboratory of Safety Evaluation for New Drugs, Zhejiang Academy of Medical Sciences, Hangzhou, Zhejiang, China; 4 Clinical laboratory, The Seventh Hospital of Ningbo, Ningbo, Zhejiang, China; 5 The Affiliated Hospital, Ningbo University, Ningbo, Zhejiang, China; University of Alabama at Birmingham, United States of America

## Abstract

The goal of our study is to investigate the contribution of promoter DNA methylation of α-adducin (ADD1) gene to the risk of essential hypertension (EH). Using the bisulphite pyrosequencing technology, DNA methylation levels of five CpG dinucleotides on *ADD1* promoter were measured among 33 EH cases and 28 healthy controls. Significantly higher *ADD1* DNA methylation levels were observed in the females than in the males (CpG1: *P* = 0.016; CpG2-5: *P* = 0.021). A breakdown analysis by gender showed that lower CpG1 methylation was associated with an increased risk of EH in females (adjusted *P* = 0.042). A much more significant association between lower CpG2-5 methylation levels and the increased risk of EH was found in males (adjusted *P* = 0.008). CpG1 methylation was inversely correlated with age in females (r = −0.407, *P* = 0.019) but not in males. *ADD1* CpG1 and CpG2-5 methylation levels were significantly lower in post-menopausal (>50 years) women than pre-menopausal (≤50 years) women (CpG1: *P* = 0.006; CpG2-5: *P* = 0.034). A significant interaction between CpG1 methylation and age was found in females (CpG1*age: *P* = 0.029). CpG2-5 methylation was shown as a significant predictor of EH in males [area under curve (AUC) = 0.855, *P* = 0.001], in contrast that CpG1 methylation was a trend toward indicator in females (AUC = 0.699, *P* = 0.054). In addition, significant differences were observed between males and females for alanine aminotransferase (ALT, *P* = 0.001), aspartate aminotransferase (AST, *P* = 0.005) and uric acid (*P*<0.001). The concentration of AST was inversely correlated with *ADD1* CpG2-5 methylation levels in female controls (r = −0.644, *P* = 0.024). These observations may bring new hints to elaborate the pathogenesis of EH.

## Introduction

Essential hypertension (EH) is one of the most important causes of premature death worldwide. EH is a complex disorder resulting from both genetic and environmental factors [1], [2]. Approximately 20–60% of the blood pressure variability in general population is heritable [3]. Epidemiological studies have documented environmental factors such as physical inactivity, obesity, high sodium and low potassium diet, and alcohol consumption are associated with hypertension risk [4], [5]. Disorders in the metabolism of high-density lipoprotein cholesterol (HDL-C) and triglycerides (TG) play a key role in EH progression [6], [7].

A sexual dimorphism exists in the developmental origins of EH [8], [9]. Males are reported to be more susceptible to hypertension than females [10]. Gender difference in the risk of hypertension was observed to be associated with altered expression of hormone receptors such as renal alpha2-adrenergic receptors [11] and angiotensin receptors [12]. In addition, evidence has shown a gender dimorphism in the expression of renin [13], [14] and urinary angiotensinogen excretion [15] which are important risk factors for EH. The sexual dimorphism in mammalian gene expression have been observed to be linked to the gender differences in the amounts of sex hormones [16] (e.g., estrogens, androgens).

*ADD1* gene encodes one of adducin subunits (α-adducin) [17]. Adducin modulates the surface expression of multiple transporters and ion pumps, and thus regulates cellular signal transduction and cytolemma ion transport [18]. Human and animal model studies have found that *ADD1* gene is a candidate gene for EH [18], [19]. However, epidemiological studies have shown that the contribution of *ADD1* Gly460Trp mutation (rs4961) to hypertension varies among different ethnic groups [20]–[24]. Meta-analyses were unable to reach a consensus for this mutation [25]–[28]. Moreover, gender dimorphism of the association between hypertension and *ADD1* Gly460Trp was observed in Caucasians [29].

DNA methylation is a stable epigenetic mark and usually occurs at cytosine residues in the context of cytosine-phosphate-guanine dinucleotide (CpG) in mammalian cells [30]. Promoter DNA methylation is linked to transcriptional silencing of protein-coding genes [31] and thus regulates the function of protein. Aberrant methylation is shown to play important roles in the occurrence and development of diseases including colorectal cancer [32], [33], breast cancer [34], [35], coronary artery disease [36] and schizophrenia [37], [38]. The evidence of the association between DNA methylation and the risk of EH was scarce. A significant decline in global DNA methylation level is observed in EH patients and the trend continues along with the progression of hypertension [39]. Altered global DNA methylation in pre-eclampsia placentas was shown to be associated with maternal hypertension [40]. Aberrant DNA methylation of *11beta-HSD2* and *Adrb1* genes were found to be associated with EH [41] and the outcome of medications [42], respectively.

We hypothesize that *ADD1* promoter DNA methylation contributes to EH. Our goal is to study whether promoter DNA methylation of *ADD1* gene is associated with EH, and to explore the interaction of promoter DNA methylation with gender and clinical indicators of lipid and amino acid metabolism.

## Materials and Methods

### Sample Collection

This study comprised 33 cases (14 males, 50.1±4.9 years; 19 females, 51.3±4.7 years) and 28 controls (14 males, 51.3±6.3 years; 14 females, 47.9±5.0 years) collected from the community residents in Zhenhai district of Ningbo city in Zhejiang province, China. All individuals are Han Chinese living in Ningbo city for at least three generations. Hypertensive patients were defined according to the golden standard [43]. All hypertensives have received antihypertensive medications for more than three months or have at least three consecutive records of systolic blood pressure (SBP) >140 mmHg and/or diastolic blood pressure (DBP) >90 mmHg (European Society of Hypertension-European Society of Cardiology Guidelines, 2003). Patients had SBP<120 mmHg and DBP<80 mmHg and had no family history of hypertension in the first degree relatives were recruited as controls. None of the controls has received antihypertensive therapy. The gender and age of controls were well matched with EH cases. All the individuals don’t have a history of diabetes mellitus, secondary hypertension, myocardial infarction, stroke, renal failure, drug abuse and other serious diseases. A calibrated mercury sphygmomanometer with appropriate adult cuff size was applied to measure blood pressures according to a standard protocol recommended by the American Heart Association [44]. Blood pressures were measured in supine position by two trained observers at an interval of at least 10 minutes. Blood samples were collected in 3.2% citrate sodium-treated tubes and then stored at −80°C for DNA extraction. The study protocol was approved by the ethical committee of Ningbo University. The informed written consent was obtained from all subjects.

### Phenotypes Collection

Blood samples were obtained after a 12 h overnight fast from the antecubital vein using vacutainer tubes containing EDTA. Plasma levels of cholesterol, TG, ALT, AST, uric acid and glucose concentrations were enzymatically measured using CX7 biochemistry analyzer (Beckman, Fullerton, CA).

### DNA Methylation Assay

**Figure 1:**
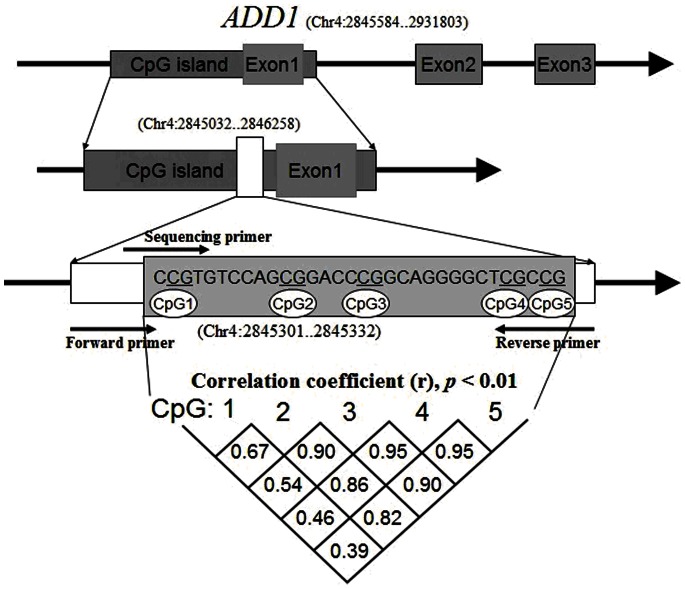
Correlation among five CpGs in *ADD1* gene promoter.

Human genomic DNA was prepared from peripheral blood samples using the nucleic acid extraction automatic analyzer (Lab-Aid 820, Xiamen City, China). DNA was quantified using the PicoGreen® double strand DNA (dsDNA) Quantification Kit (Molecular Probes, Inc. Eugene, USA). Bisulphite pyrosequencing technology was used to determine the 5 CpG dinucleotides methylation levels on the fragment within *ADD1* promoter (Figure 1). Pyrosequencing assays combine sodium bisulfite DNA conversion chemistry (EpiTech Bisulfite Kits; Qiagen; #59104), polymerase chain reaction (PCR) amplification (Pyromark PCR Kit; Qiagen; #978703) and sequencing by synthesis assay (Pyromark Gold Q24 Reagents; Qiagen; #978802) of the target sequence. Sodium bisulfite preferentially deaminates unmethylated cytosine residues to thymines (after PCR amplification), whereas methyl-cytosines remain unmodified. PCR primers were selected using PyroMark Assay Design software v2.0.1.15. The PCR and pyrosequencing primers for *ADD1* gene promoter amplification were described in Table S1.

### Statistical Analysis

Statistical analyses were performed to investigate the association among *ADD1* DNA methylation, metabolic profile and EH. Either Pearson chi-square or Fisher exact test was used for the association of EH with categorical variables including gender, smoking, and drinking. Two sample *t*-test was applied for the association of EH with continuous variables including age, body mass index (BMI), total cholesterol, total triglycerides, glucose, ALT, AST, and uric acid. Pearson correlation was used to determine the association between the *ADD1* DNA methylation and metabolic characteristics. Receiver operating characteristic (ROC) curve was used to analyze the sensitivity of *ADD1* DNA methylation in EH diagnosis. Logistic regression was implemented for the interaction of *ADD1* methylation and age. A two-sided p-value <0.05 was considered statistically significant. All the above statistical analyses were performed with PASW Statistics 18.0 software (SPSS, Inc., Somers, NY, USA). Meanwhile, Power and Sample Size Calculation software (v3.0.43) was used to estimate the power of the study [45].

## Results

A total of 33 cases and 28 age- and gender-matched controls were recruited in the current association study. As shown in Table 1, mean levels of body mass index (BMI) and all metabolic phenotypes were within normal ranges. The mean levels of age and BMI were well paired between males and females (Table 1).

**Table 1:** Characteristics of all subjects (n = 61).

Characteristics	Mean ± s.e.	Men (Mean ± s.e.)	Women (Mean ± s.e.)	p_gender_	EH (Mean ± s.e.)	Non-EH (Mean ± s.e.)	p_EH_
Age (years)	50.2±5.3	50.7±5.5	49.8±5.1	0.527	50.8±4.7	49.6±5.8	0.361
Gender (M/F)	28/33	NA^c^	NA^c^		14/19	14/14	0.554
BMI (kg/m^2^)^a^	22.50±2.55	22.63±1.77	22.41±3.01	0.781	22.95±2.72	21.84±2.19	0.159
Smoking (Y/N)	NA^c^	9/19	0/33	0.002	5/28	3/25	0.896
Drinking (Y/N)	NA^c^	6/22	0/33	0.018	4/29	2/26	0.826
Total cholesterol (mmol/L)	5.05±0.93	5.01±0.85	5.08±1.01	0.775	5.09±0.81	4.99±1.07	0.681
Total triglycerides (mmol/L)	1.50±0.91	1.44±0.84	1.56±0.97	0.615	1.69±1.08	1.28±0.58	0.066
Glucose (mmol/L)	5.18±0.58	5.15±0.59	5.20±0.58	0.735	5.31±0.59	5.02±0.53	0.055
ALT (IU/L)	21.4±15.5	28.6±17.9	15.3±9.9	0.001	23.7±13.5	18.8±17.5	0.228
AST (IU/L)^b^	23.9±7.0	26.6±7.7	21.5±5.4	0.005	25.3±5.7	22.2±8.3	0.089
Uric Acid (µmol/L)	298.8±88.8	351.5±91.0	254.0±57.3	0.000	312.0±96.4	283.2±77.7	0.210
CpG1 methylation (%)	9.97±2.24	9.25±1.40	10.58±2.63	0.016	9.52±1.46	10.50±2.85	0.091^d^
CpG2-5 methylation (%)	29.33±6.81	27.17±7.56	31.16±5.58	0.021	27.54±7.48	31.44±5.30	0.026^d^

a n = 44 (18 men versus 26 women, 26 EH versus 18 Non-EH);

b n = 59 (28 men versus 31 women, 33 EH versus 26 Non-EH).

c NA denotes not applicable.

d The p-values were adjusted for age, gender, smoking and drinking using a logistic regression analysis.

**Figure 2:**
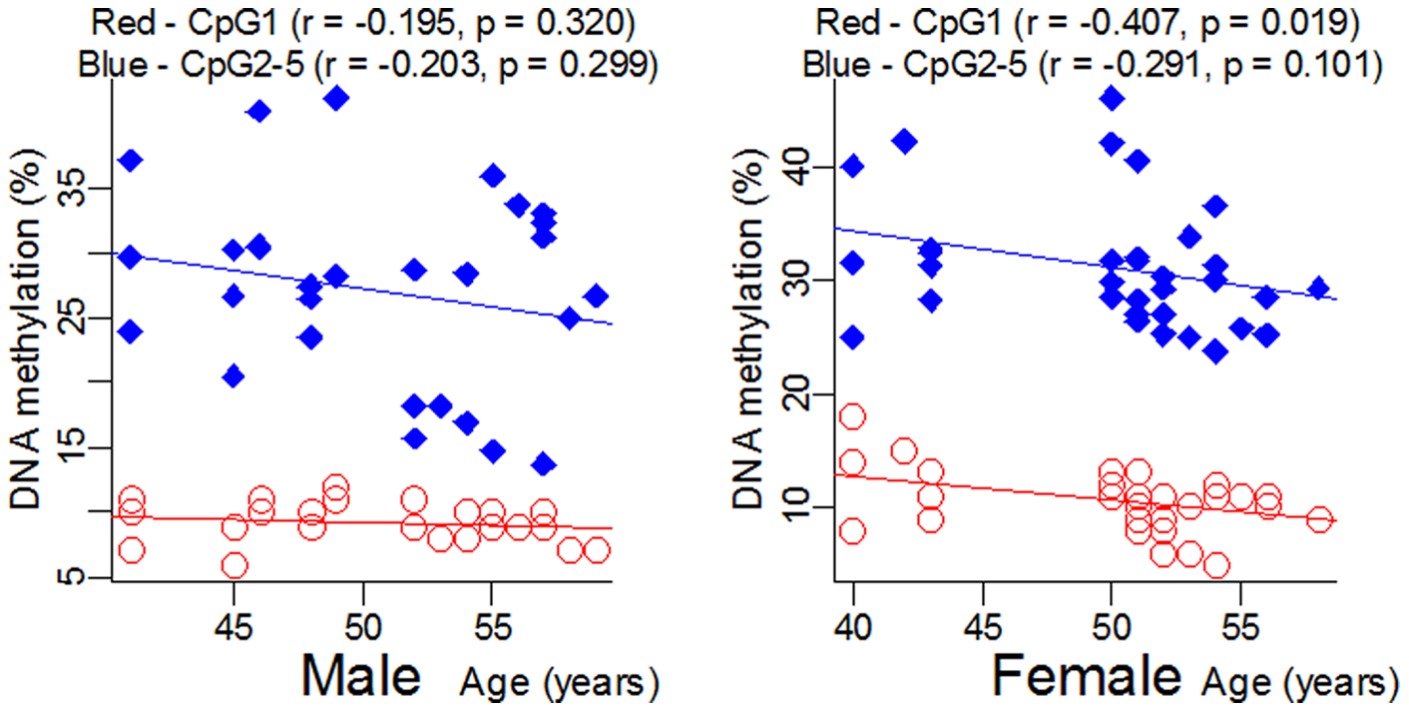
Pearson correlation between *ADD1* methylation and age in males and in females.

**Figure 3:**
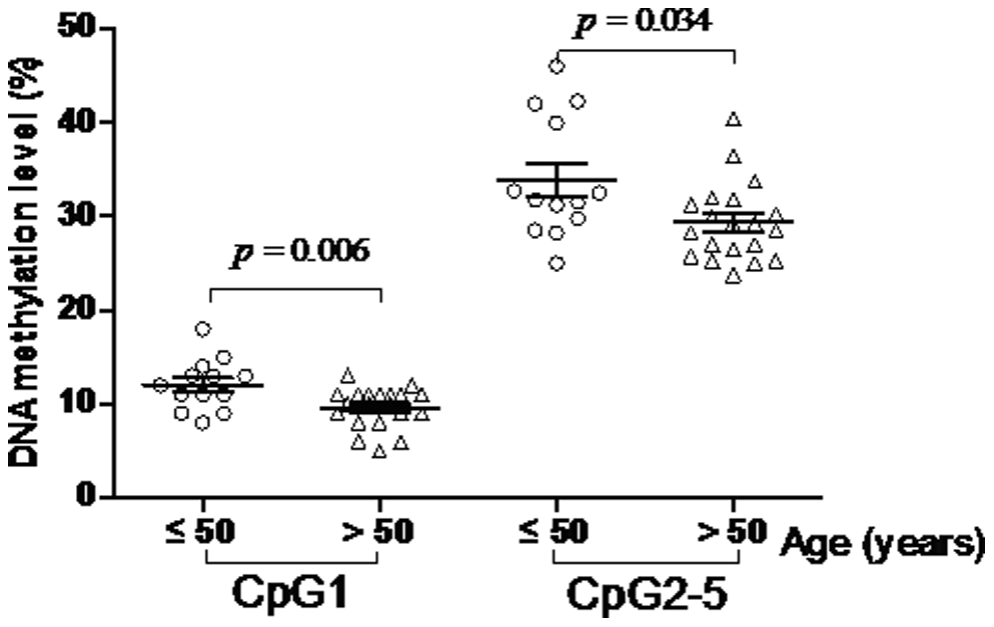
Significant *ADD1* methylation difference between pre-menopausal and post-menopausal females.

In this study, we selected a locus containing 5 CpG dinucleotides to explore the association of *ADD1* gene promoter DNA methylation with EH (Figure 1). We found DNA methylation levels were closely correlated among CpG2-5 (Figure 1, r >0.80, *P*<0.001). As shown in Table 1 and Figure S1, our results showed that *ADD1* CpG2-5 methylation levels were significantly associated with EH (cases versus controls (%): 27.54±7.48 versus 31.44±5.30, adjusted *P* = 0.026). Moreover, significantly higher *ADD1* DNA methylation levels were observed in females than in males (CpG1: *P* = 0.016; CpG2-5: *P* = 0.021). CpG1 methylation was inversely correlated with age in females (Figure 2, r = −0.407, *P* = 0.019). *ADD1* methylation levels were significantly higher in pre-menopausal (≤50 years) women than post-menopausal (>50 years) women (Figure 3, CpG1: *P* = 0.006; CpG2-5: *P* = 0.034).

**Figure 4:**
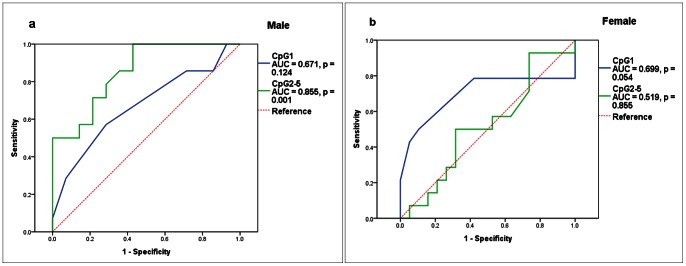
ROC Cure of *ADD1* promoter DNA methylation in EH (a, b).

A breakdown association test by gender was also performed to explore the association between *ADD1* methylation levels (including CpG1 and CpG2-5) and EH. As shown in Table 2 and Figure S1, *ADD1* CpG1 methylation level was significantly associated with EH in females (cases versus controls (%): 10.00±1.41 versus 11.36±3.63, adjusted *P* = 0.042) but not in males (adjusted *P* = 0.133). As shown in Table 2 and Figure S1, lower levels of *ADD1* CpG2-5 methylation were associated with increased risk of EH in males (cases versus controls: 22.48% versus 31.86%, adjusted *P* = 0.008). In contrast, no association of CpG2-5 methylation levels with EH was found in females (adjusted *P* = 0.557). Furthermore, significant interaction of CpG1 methylation and age was found to influence EH status in females (CpG1*age: *P* = 0.029). Prediction potential of EH for *ADD1* CpG1 and CpG2-5 methylation levels was assessed by the ROC curves. CpG2-5 methylation was shown as a significant predictor of EH in males [Figure 4a, area under curve (AUC) = 0.855, *P* = 0.001]. CpG1 methylation was also shown with a trend toward indicator in females (Figure 4b, AUC = 0.699, *P* = 0.054).

**Table 2:** Characteristics comparison between EH and Non-EH stratified by gender.

Characteristics	EH Mean± s.e.	Non-EH Mean ± s.e.	p value
**Men (28)**			
Age (years)	50.1±4.9	51.3±6.3	0.595
BMI (kg/m2)^a^	22.70±2.09	22.53±1.41	0.846
Smoking (Y/N)	6/8	3/11	0.420
Drinking (Y/N)	4/10	2/12	0.648
Total cholesterol (mmol/L)	5.09±0.80	4.93±0.92	0.630
Total triglycerides (mmol/L)	1.54±0.98	1.34±0.71	0.546
Glucose (mmol/L)	5.31±0.60	4.99±0.55	0.146
ALT(IU/L)	30.4±13.9	26.9±21.6	0.607
AST(IU/L)	28.0±4.6	25.2±9.9	0.351
Uric Acid (µmol/L)	375.0±107.3	328.1±67.3	0.178
CpG1 methylation (%)	8.86±1.29	9.64±1.45	0.133^d^
CpG2-5 methylation (%)	22.48±6.29	31.86±5.65	0.008^d^
**Women (n = 33)**			
Age (years)	51.3±4.7	47.9±5.0	0.051
BMI (kg/m2)^b^	23.11±3.11	21.29±2.60	0.137
Smoking (Y/N)	0/19	0/14	
Drinking (Y/N)	0/19	0/14	
Total cholesterol (mmol/L)	5.09±0.85	5.06±1.23	0.914
Total triglycerides (mmol/L)	1.81±1.17	1.22±0.44	0.058
Glucose (mmol/L)	5.31±0.60	5.06±0.54	0.235
ALT (IU/L)	18.7±11.2	10.8±5.2	0.012
AST (IU/L)^c^	23.4±5.6	18.7±3.6	0.008
Uric Acid (µmol/L)	265.6±53.4	238.2±60.6	0.180
CpG1 methylation (%)	10.00±1.41	11.36±3.63	0.042^e^
CpG2-5 methylation (%)	31.26±6.04	31.02±5.11	0.557^e^

a n = 18 (10 EH versus 8 Non-EH);

b n = 26 (16 EH versus 10 Non-EH);

c n = 31 (19 EH versus 12 Non-EH);

d The p-values were adjusted for age, smoking and drinking using logistic-regression analysis.

e The p-values were adjusted for age, smoking, drinking, ALT and AST using logistic-regression analysis.

**Figure 5:**
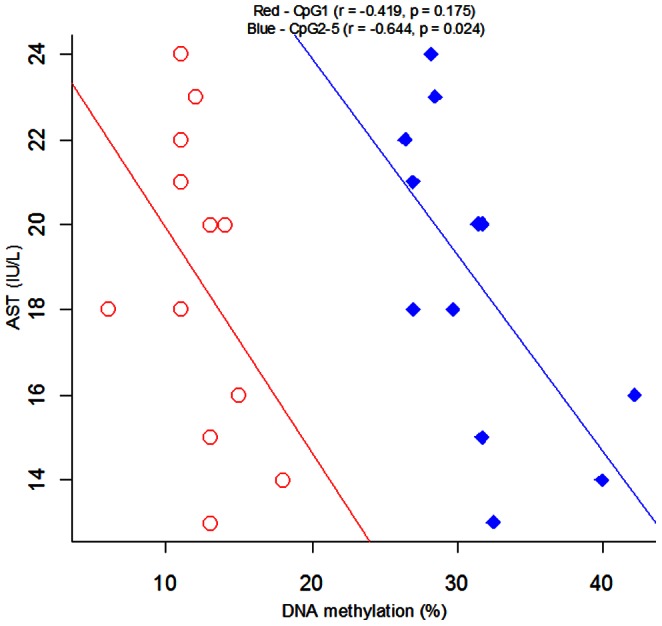
Pearson correlation between *ADD1* methylation and AST in females.

As shown in Table 1, significant differences were observed between males and females for ALT (*P* = 0.001), AST (*P* = 0.005) and uric acid (*P*<0.001). Subsequently, we performed correlation tests between *ADD1* DNA methylation levels and metabolic phenotypes including uric acid, ALT, and AST in controls. However, we didn’t find any correlations in controls between *ADD1* DNA methylation levels and these metabolic phenotypes (*P*>0.05, Figure S2a–c). A post hoc analysis was performed for the correlation between *ADD1* CpG1 and CpG2-5 methylation levels and metabolic phenotypes (uric acid, ALT, and AST) in females and males. Other than a significant correlation between CpG2-5 and AST in females (r = −0.644, *P* = 0.024, Figure 5), no significant results observed in the rest of correlation tests (Figure S3a–e).

## Discussion

The goal of the current study is to evaluate the contribution of *ADD1* gene promoter DNA methylation to EH. We found that DNA methylation of *ADD1* gene was significantly higher in females than in males. In addition, DNA methylation was shown to be a risk factor of EH in males (CpG2-5) and females (CpG1). To our knowledge, this is the first study showing the association of *ADD1* gene promoter DNA methylation with EH. Our comprehensive analysis on the role of *ADD1* methylation in the risk of EH may provide new hints to clarify the pathogenesis of EH in future.

Adducin was implicated in the pathogenesis of EH by modulating Na^+^-K^+^-ATPase activity [46]–[48]. Evidence indicated that adducin might be a candidate protein to explain genetic alterations in ion transport associated with EH [47]. A previous study reported that hypertensive rat had an increased activity and expression of Na^+^-K^+^-pump [48]. In this study, we hypothesized that the aberrant *ADD1* methylation may cause hypertension. We speculated that lower *ADD1* methylation led to higher expression of α-adducin which resulted in an increased activity and expression of Na^+^-K^+^-pump and eventually caused high Na^+^-reabsorption and hypertension. In accordance with the speculation, we did observe a significant lower level of *ADD1* gene promoter methylation in EH cases than in controls.

Interestingly, CpG1 methylation was associated with EH in females, while the CpG2-5 methylation was significantly associated with EH in males. We also found *ADD1* methylation was significantly higher in females than in males. In the present study, all subjects were recruited from Han Chinese residents in Ningbo city for at least three generations and diagnosed by generally recognized protocol. The gender and age were well matched between EH cases and Non-EH controls. The power of our association test of EH was 86.5% for *ADD1* CpG2-5 in all samples, 66.7% for *ADD1* CpG1 in females, and 84.4% for *ADD1* CpG2-5 in males. Additionally the power of the analysis stratified by menopausal status was 71% for *ADD1* CpG1 and 61.7% for *ADD1* CpG2-5. However, our study was only involved with 61 samples and we could not exclude a possibility of spurious association due to the hidden structures in the tested samples. Future replication study with larger size of samples is warranted to confirm our findings.

Sexual dimorphism was found in the whole genome analysis for the risk loci of hypertension in rats [49], [50] and humans [51]. Gender dimorphism was also observed in the association studies of hypertension. These studies addressed *ADD1* Gly460Trp polymorphism in female Caucasians [29], *CYP19A1* polymorphisms (rs700518, rs10046 and rs4646) in male Japanese [52], *SELE* T1559C polymorphism [53] and *PNMT* G390A polymorphism [54] in male Chinese, and other *P2RY2* polymorphism (rs4944831) [55]. In the current study, we found that ALT and AST were associated with EH only in females, and that *ADD1* CpG1 and CpG2-5 methylation levels were associated with EH in females and males, respectively. Moreover, CpG2-5 was correlated with AST in females, but not in males. These results may be associated with the difference of sex hormones. The role for gender (and/or sex hormones) has been described in mediating differential epigenetic effects on cardiomyocytes [56], effects of radiation [57], [58] and the endocrine system [59]. Significant higher *ADD1* methylation levels were found in pre-menopausal women than in post-menopausal ones. Our results provide new clues to explain the sexual dimorphism of EH.

DNA methylation level in humans is reported to alter along with changes of environmental factors such as nutrients [60] and drugs [61], [62]. Therefore, major risk factors for hypertension including physical inactivity, high sodium diet, alcohol consumption and obesity [4], [5] may alter the DNA methylation levels of EH risk genes and cause EH over time. Since lifestyle factors such as smoking, drinking, and physical activity are different between males and females, our findings of gender-dependent *ADD1* methylation may reflect the difference in these non-heritable risk factors of EH.

Uric acid was reported positively correlated to the incidence of EH [63]. Previous study also showed uric acid was the risk factor associated with the mortality in hypertensive stroke patients [64]. In addition, uric acid has been reported associated with the prevalence of chronic kidney disease [65]. Raised plasma ALT level was associated with hypertension in Hong Kong Chinese [66]. Another study also reported that elevated ALT and (or) AST were associated with increased risk of pulmonary arterial hypertension [67]. However, ALT and AST have mostly been reported to be associated with hepatic disease [68]–[70], and they were seldom reported to related with EH. In the current study, we didn’t find the associations of uric acid and ALT with *ADD1* DNA methylation levels in controls, whereas CpG2-5 was associated with AST in females. Future study is needed to investigate the mechanism underlining this association.

In summary, this study presents a lower *ADD1* promoter methylation increases EH risk. *ADD1* CpG2-5 methylation is able to predict EH risk in males with higher fidelity than CpG1 methylation does in females. The loss of power for CpG1 may be due to a significant interaction between CpG1 and age in females. Our findings are likely to bring new hints to elaborate the pathogenesis of this complex disease.

## Supporting Information

Figure S1 **Subgroup analysis in** ***ADD1*** **promoter DNA methylation^a^.** a: Triangles and circles stand for males and females respectively; blue and red stand for cases and controls, respectively.
(TIF)

Figure S2 **Pearson correlation between** ***ADD1*** **methylation and metabolic phenotypes in controls (A–C).**
(TIF)

Figure S3 **Pearson correlation between** ***ADD1*** **methylation and metabolic phenotypes in males (A–C) and in females (D–F).**
(TIF)

Table S1 **Primers for** ***ADD1*** **gene CpG island loci analysis.**
(DOC)
