# Supplementary figures and images for: Lower ADD1 Gene Promoter DNA Methylation Increases the Risk of Essential Hypertension
Source: PLoS One. 2013 May 15;8(5):e63455. doi: 10.1371/journal.pone.0063455 (PMC3655193; doi:10.1371/journal.pone.0063455)

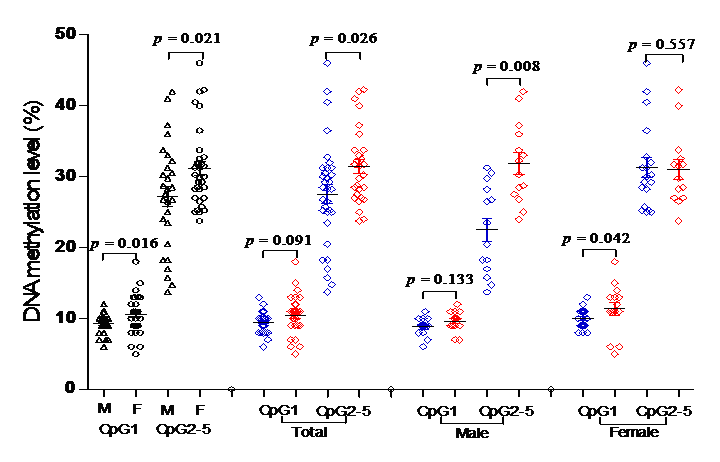

Supplement: Figure S1 — Subgroup analysis in ADD1 promoter DNA methylationa. a: Triangles and circles stand for males and females respectively; blue and red stand for cases and controls, respectively. (TIF) [file pone.0063455.s001.tif]

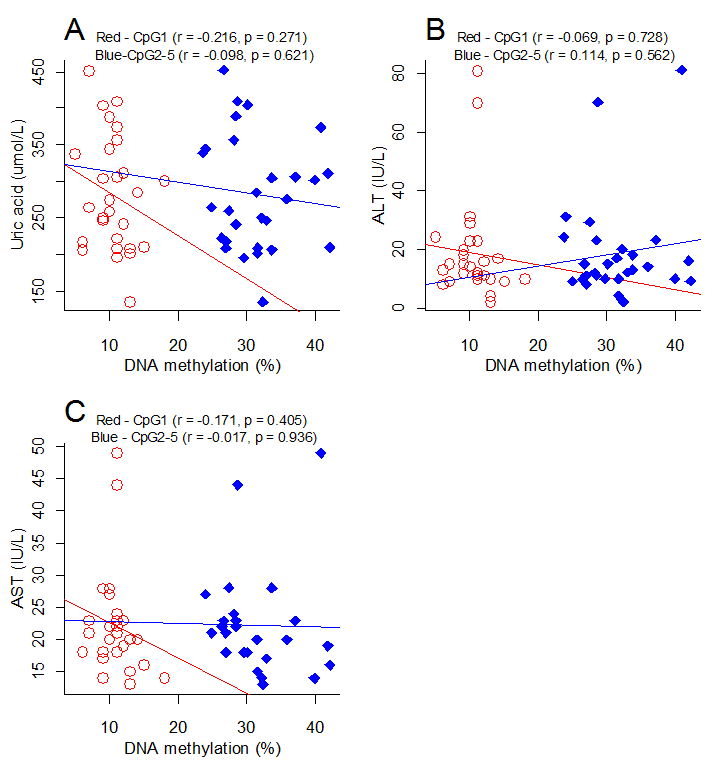

Supplement: Figure S2 — Pearson correlation between ADD1 methylation and metabolic phenotypes in controls (A–C). (TIF) [file pone.0063455.s002.tif]

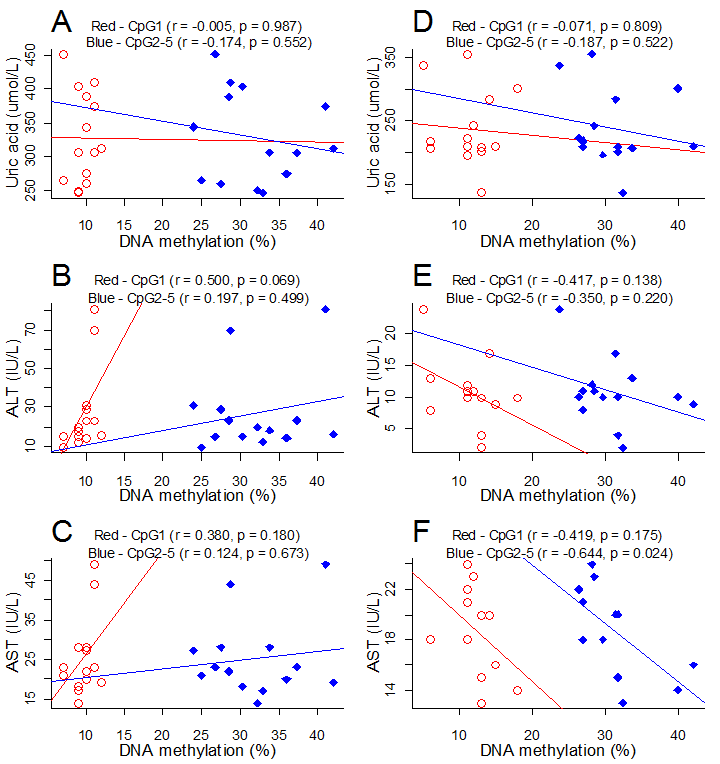

Supplement: Figure S3 — Pearson correlation between ADD1 methylation and metabolic phenotypes in males (A–C) and in females (D–F). (TIF) [file pone.0063455.s003.tif]
