# Supplementary material for: Lower ADD1 Gene Promoter DNA Methylation Increases the Risk of Essential Hypertension
Source: PLoS One. 2013 May 15;8(5):e63455. doi: 10.1371/journal.pone.0063455 (PMC3655193; doi:10.1371/journal.pone.0063455)
Supplement: Table S1 — Primers for ADD1 gene CpG island loci analysis. (DOC) [file pone.0063455.s004.doc]

Table S1: Primers for *ADD1* gene CpG island loci analysis

| Group | DNA Sequence |
| --- | --- |
| Forward primer | 5’-AAAATTAGGTTTGGGGATTGTATAAGG-3’ |
| Reverse primer | 5’-Biotin-CCAAATCATAACTTCACCATAACCAAATAAAC-3’ |
| Sequencing primer | 5’-TTTTAGGAGGAGGTTAATTATAATG-3’ |
